# Health-related quality of life in diabetic patients and controls without diabetes in refugee camps in the Gaza strip: a cross-sectional study

**DOI:** 10.1186/1471-2458-6-268

**Published:** 2006-10-30

**Authors:** Ashraf Eljedi, Rafael T Mikolajczyk, Alexander Kraemer, Ulrich Laaser

**Affiliations:** 1 Faculty of Nursing, The Islamic University of Gaza, Gaza strip, Palestinian Territories; 2 Department of Public Health Medicine, School of Public Health, University of Bielefeld, Bielefeld, Germany; 3 Department of Epidemiology and International Public Health, School of Public Health, University of Bielefeld, Bielefeld, Germany

## Abstract

**Background:**

Prevalence of diabetes mellitus is increasing in developed and developing countries. Diabetes is known to strongly affect the health-related quality of life (HRQOL). HRQOL is also influenced by living conditions. We analysed the effects of having diabetes on HRQOL under the living conditions in refugee camps in the Gaza strip.

**Methods:**

We studied a sample of 197 diabetic patients who were recruited from three refugee camps in the Gaza strip and 197 age- and sex-matched controls living in the same camps. To assess HRQOL, we used the World Health Organization Quality of Life questionnaire (WHOQOL-BREF) including four domains (physical health, psychological, social relations and environment). Domain scores were compared for cases (diabetic patients) and controls (persons without diabetes) and the impact of socio-economic factors was evaluated in both groups.

**Results:**

All domains were strongly reduced in diabetic patients as compared to controls, with stronger effects in physical health (36.7 vs. 75.9 points of the 0–100 score) and psychological domains (34.8 vs. 70.0) and weaker effects in social relationships (52.4 vs. 71.4) and environment domains (23.4 vs. 36.2). The impact of diabetes on HRQOL was especially severe among females and older subjects (above 50 years). Low socioeconomic status had a strong negative impact on HRQOL in the younger age group (<50 years).

**Conclusion:**

HRQOL is strongly reduced in diabetic patients living in refugee camps in the Gaza strip. Women and older patients are especially affected.

## Background

Diabetes mellitus is a serious disease and a cause for a growing public health concern in both developed and developing countries. Globally, the number of people with diabetes is expected to double between 2000 and 2030 while public awareness about this disease remains low [1,2]. Several studies have demonstrated that diabetes has a strong negative impact on the health-related quality of life (HRQOL), especially in the presence of complications [3-9]. However, most of the studies on diabetes and HRQOL have been conducted in developed countries (review in [10]) and studies of the HRQOL in diabetic patients in developing countries are rare [11-14].

In the Gaza strip, more than one third of the Palestinian population lives in eight very densely populated camps [15]. The prevalence of diabetes was estimated at 11% in the rural and 14% in the urban population of the West-Bank [16]. The prevalence of complications of diabetes in this population is high [17] and complications of diabetes are known to negatively affect the HRQOL of diabetic patients. In an Iranian randomized trial a health education programme considerably improved the HRQOL of diabetic patients in the intervention group [14]. However, because of the lack of controls without diabetes living under the same conditions, the authors were not able to assess to which extent the intervention reduced the gap between diabetic patients and healthy controls. In a population-based Dutch study diabetic patients without complications had only slightly lower HRQOL than age matched persons in the general population [4]. However, the effects of socio-economic differences may be weaker in developed countries than in conditions of poverty in developing countries. The negative impact of socio-economic conditions on HRQOL among diabetic patients can be even stronger in refugees. The aim of our study was to examine the HRQOL of diabetic patients in a large and long-term refugee community in camps in the Gaza strip in comparison to controls living in similar conditions. We focussed on the question whether variables potentially affecting HRQOL in healthy controls have a different impact in diabetic patients. The secondary aim of the analysis was to evaluate the psychometric properties of the Arabic version of a HRQOL instrument.

## Methods

### Sample

For the purpose of comparison we aimed at recruiting about 200 patients with diabetes and 200 controls without diabetes living in the camps in the Gaza strip. We started with diabetic refugees who were randomly selected from the files in "United Nations Relief and Works Agency for Palestine" clinics (UNRWA) in the biggest three refugee camps in the Gaza strip: Jabalia in the North, Nuseirat in the Middle, and Rafah in the South. The sample obtained at each site was proportional to the population living in the respective regions. Inclusion criteria for patients were: being a resident of one of the three refugee camps, a diagnosis of type 1 or type 2 diabetes with treatment initiated at least six months prior to the study. Exclusion criteria were having any co-morbidities (any chronic disease not clearly related to diabetes, patients with complications of diabetes were not excluded) and pregnancy at the time of the survey. The distinction between co-morbidities and complications of diabetes was based on medical records of the patient. Diabetes was diagnosed according to any one of the following criteria: (1) symptoms of diabetes and random plasma glucose concentration ≥ 200 mg/dl (11.1 mmol/l), (2) fasting plasma glucose ≥ 126 mg/dl (7.0 mmol/l), (3) 2-hours plasma glucose ≥ 200 mg/dl (11.1 mmol/l) during an oral glucose tolerance test.

The first 20 patients were recruited for pilot-testing the survey and were therefore excluded from the main study. Of the following 299 patients with diabetes, 67 suffered from other diseases and 7 were pregnant, leaving 225 eligible patients for participation. 28 patients refused to participate, resulting in a final sample of 197 (response rate of 88%). Through referrals by diabetic patients we recruited 197 refugees without diabetes or any other chronic disease living in the same camps that were matched to patients by age groups and gender. For logistic reasons the number of controls refusing participation could not be obtained. All participants were asked to complete a self-administered questionnaire. Data were collected from November 2003 to December 2004. Written informed consent was obtained from all participants. The study was approved by the Ministry of Health and the United Nations Health Office in the Gaza strip.

### Questionnaire

We used the World Health Organization Quality of Life Questionnaire – short version (WHOQOL-BREF) to assess quality of life [18,19]. This questionnaire was developed with 15 international field centres to obtain an assessment tool that is applicable cross-culturally. It was used in 212 publications currently referenced in Medline (search term: "WHOQOL", April 2006), 4 of them dealing with diabetic patients (search term: "WHOQOL diabetes"). The four domains of the WHOQOL-BREF are physical health, psychological (e.g. self-esteem), social relationships (e.g. social support) and environment (e.g. freedom, physical safety). Subjects rated all items on a 5 point Likert-type scale. The questionnaire was translated into Arabic language using two independent translations from English to Arabic. The translations were reviewed by the first author (A.E.) and synthesised into one version. This preliminary Arabic questionnaire was back-translated into English by two independent translators. Both versions were compared and the Arabic version was revised where necessary.

Raw scores for the domains of WHOQOL-BREF were calculated by adding values of single items and were transformed on the scale ranging from 0 to 100, where 100 is the highest and 0 the lowest HRQOL [20]. Additionally to the variables used for matching (gender, age and place of residence) we also collected some limited socio-demographic (marital status, education, own housing versus rental, occupational status) and disease related information (type of diabetes, duration of diabetes since the first diagnosis, medication for diabetes, complications of diabetes).

### Statistical analysis

We evaluated the psychometric characteristics of the Arabic translation of WHOQOL-BREF in the sample by means of internal reliability (Cronbach's alpha). We used the chi-square test for comparison of categorical variables and the t-test for the comparison of mean score values for the domains of WHOQOL-BREF between groups. Further, we performed multivariate analysis of variance to analyse the determinants of HRQOL in both groups. All independent variables were dichotomised. We followed standard recommendations by Hosmer and Lemeshow for model-building strategies [21]. Since most of the variables had a significant interaction with the age and case-control status including them all jointly in the model would yield too many variables per observation. We therefore included interactions in two separate sets: interactions between case-control status and all other variables and interactions between age groups and all other variables. From each set we eliminated interactions which did not cross the significance level of 0.1. Finally, we included all interactions from the previous step in the model and eliminated stepwise all terms which were not significant on the 0.05 level. Based on the final model we calculated the contribution of interactions to the HRQOL with Wald-type confidence intervals, comparing to a single reference category for all strata. All analyses were performed with SPSS^® ^for Windows version 12, for the analysis of variance we used the GLM procedure and obtained the significance statistics from multivariate tests including all domains using the Pillai-Spur criterion. The choice of multivariate technique was motivated by a potential correlation of domain scores within the respondents and had the benefit of a homogenous set of independent variables in the models for all outcomes despite using variable selection. The interpretation of the results is based on a clinically meaningful difference of 5 points of the 0–100 scores.

## Results

### Psychometric characteristics of the WHOQOL-BREF in the study sample

The internal reliability was very high for three domains and reasonably high for the fourth one. Cronbach's alpha for the physical health domain was 0.95, for the psychological domain 0.94, for social relationships domain 0.6 and 0.85 for the environment domain. For the physical health, psychological and environment domains, the internal reliabilities could not be improved through removing any of the items. In the social relationships domain, the item related to sexual life satisfaction had a considerably lower mean than the remaining items: personal relationships and support from friends. Removing this item from the scale would increase internal reliability. In the analysis stratified by gender this observation persisted, however, stratifying by case-control status (diabetes versus non-diabetes) this was true only for diabetic patients. For the purpose of standardisation we used the instrument as given and did not remove the sexual life satisfaction item from the social relationships domain.

### Characteristics of the sample

Socio-economic information for the two groups is presented in Table 1. Patients with diabetes were more often widowed, had lower levels of education and income, but were more often employed than non-diabetic controls. They were mostly patients with type 2 diabetes (92.4%) with a relatively long history of disease (72.6% over 6 years, 33.5% over 10 years). Despite the high proportion of patients with complications (80.2% with one or more self-reported major complication: nephropathy, neuropathy, retinopathy or diabetic foot), more than half were treated by oral medication (53.8%). Female patients had a longer duration of the disease and more self-reported complications than male patients (Table 2).

### Evaluation of the HRQOL

The HRQOL was significantly lower for diabetic patients than for controls in all of the domains with the largest differences in the physical health and psychological domains (39 and 35 points difference) and smaller differences in social relationships and environment domains (19 and 13 points difference) (Table 3). Both groups had particularly low scores in the environmental domain with the smallest difference between groups, indicating the bad environmental conditions affecting HRQOL of diabetic patients and controls in a similar way.

In the multivariate analysis (joint analysis for all domains) several interactions including group status (diabetic patients vs. controls) and age were significant (p < 0.05) (Table 4). Female diabetic patients had consistently lower HRQOL for all domains than male diabetic patients, whereas the gender difference in controls was negligible. For example healthy women reported a 0.5 point better score in the physical health domain, but women with diabetes a 9.1 (-45.2-(-36.1) = 9.1) point lower score. Similarly, diabetic patients younger than 50 years had a considerably better HRQOL than older patients, whereas the age effect in controls was very small. Diabetic patients with a lower number of persons financially depending on them had a decreased HRQOL, but non-diabetic patients with a lower number of persons financially depending on them had a slightly increased HRQOL. Lower income (below 200 $) had stronger effect on HRQOL among patients younger than 50 than for older patients. In the age group below 50 years, being in the category with lowest income was comparable to having diabetes in terms of impact on physical health and psychological domain. The socio-economic status had even a higher impact on social relationships and environment domain in this comparison. Being married had a small positive effect on HRQOL.

## Discussion

We analysed the HRQOL in a sample of diabetic patients living in refugee camps in the Gaza strip in comparison to gender- and age-matched non-diabetic controls from the same camps. Diabetes and its complications affected negatively all of the domains of the WHOQOL-BREF, however the effects were strongest for the physical health and psychological domains and weaker for the social relationships and environment domains. We found a strong effect of interactions between gender and disease status (diabetic patients vs. controls). Whereas this finding could be partly explained by the worse situation of female patients in respect to the disease in our sample, this is still an evidence for gender inequalities. Similar difference in HRQOL of both genders was observed in a study conducted in Iran [13]. Lower HRQOL in women with diabetes was also reported in other studies [4,5]. Age strongly affected the HRQOL of diabetic patients in physical health and psychological domains and had almost no effects on HRQOL among controls. Had we not taken into account the interactions, we would have found mistakenly that these factors also severely impact HRQOL among controls. Interestingly, in the analysed set of variables, education played no independent role with respect to HRQOL. Another important finding is the high impact of the economic situation on the HRQOL (which did not interact with the disease status), especially in the younger age group. This is a big concern, because more than one half of the sample had an income in the lowest category.

This study also contributed to the literature by translating the WHOQOL-BREF into Arabic and by administering the assessment tool for the first time to an Arabic speaking sample. The psychometric properties of the Arabic translation of the WHOQOL-BREF were somewhat better than in a Norwegian general population sample [22] or Danish sample consisting of five different groups of patients [23], but this may be due to the fact that our sample was more homogenous.

The strengths of this study are the relatively large sample size and inclusion of a control group. Further, the sample was designed to represent the refugee population in the Gaza strip camps, a population which is not only underserved but also understudied. There are however several weaknesses of this study. Diabetic patients recruited for this study are a random sub-sample of patients treated in the UNRWA clinics but may not represent all diabetic patients living in the camps. Whereas this restriction does not threaten the internal validity of the analysis, findings may be not generalisable. The Palestinian Health care system comprises three sectors: the governmental, UNRWA, and the private sector. Most of the refugees in the camps receive care fromthe UNRWA, but some of the refugees are able to pay for the health insurance and they prefer to seek health care from the governmental institutions because of their comprehensiveness. Other refugees receive health care from the private sector outside of the camps. Patients treated at the UNRWA clinics may have a worse health condition than patients receiving care from other providers. In some cases the medical records regarding complications of diabetes may have been incomplete. This information was not verified by an examination by the physician for the purpose of this study. This may have resulted in underreporting of complications, but this does not affect the comparison between diabetic patients and controls.

We do not know whether the controls proposed by the patients had any characteristics which were related to either higher or lower HRQOL than in the general population. The most striking difference between diabetic patients and controls was their level of education. The most likely explanation is that this reflects the lower willingness of controls with lower education to participate in the study. However, the number of eligible controls who refused participation was not appropriately recorded to provide evidence for this assumption. Given the practical limitations, the sampling technique for controls appeared most appropriate and was successfully implemented in other studies [24].

In a Danish validation study of the WHOQOL-BREF the mean scores were considerably higher for all the domains in diabetic patients (between 70 and 76 points) as compared to our sample (23 to 52), but only slightly higher for controls (74 to 89 versus 70 to 76) with the exception of the environment domain, where the score in our sample was much lower (80 vs. 36) [23]. In the Iranian study the scores for the diabetic patients were lower than in the Danish study (55 to 65) but still considerably higher than in our sample [13]. Still the largest difference between our data and other studies was for the environment domain.

## Conclusion

Diabetic patients living in the Gaza strip camps have a poor HRQOL compared to non-diabetic controls living under the same conditions and diabetic patients surveyed in other studies. Higher age further reduces the HRQOL in diabetic patients without having an impact on controls. Diabetes has a greater impact on the HRQOL of females than on the HRQOL of males. Under the living conditions encountered in this study, poverty strongly reduced HRQOL, especially in younger participants and this effect did not differ for diabetic patients and controls.

## Competing interests

The author(s) declare that they have no competing interests.

## Authors' contributions

AE planned and conducted the study and drafted the first version of the manuscript. RTM performed the statistical analysis and wrote the final version of the manuscript. UL and AK contributed to study design and conceptualization. All authors read and approved the final manuscript.

## Pre-publication history

The pre-publication history for this paper can be accessed here:



## Figures and Tables

**Table 1:** Socio-economic characteristics of the sample (%)

Characteristic	Diabetic refugees N = 197	Non-diabetic refugees N = 197	p-value Chi-square
*Matching factors*			
Gender			-
Male	52.8	52.8	
Female	47.2	47.2	
Age groups			-
<30	6.1	6.1	
30–39	10.7	10.7	
40–49	24.9	24.9	
50–60	33.5	33.5	
> 60	24.9	24.9	
*Other variables*			
Education			<0.001
None	28.4	4.1	
Primary	14.7	4.11	
Secondary	15.2	13.7	
Tertiary	20.3	35.0	
Higher	21.3	43.1	
Marital status			0.001
Single	7.1	8.6	
Married	76.6	87.3	
Widowed	17.7	3.6	
Divorced	1.5	0.5	
House type			0.26
Owned	69.5	68.5	
Refugee shelter*	24.5	22.3	
Rented	5.1	9.1	
Monthly Income			0.001
Below 200 US$	65.5	50.8	
200–500 US$	24.9	42.1	
>500 US$	9.6	7.1	
Employment			<0.001
No	59.4	66.5	
Yes	40.6	33.5	
Number of people financially depending on the respondent			0.003
0–5	58.9	61.9	
6–10	38.1	27.4	
> 10	3.0	10.7	

* The temporary houses built by the United Nations Relief and Works Agency for Palestinian Refugees in the Near East (UNRWA) in 1948 as the first shelter.

**Table 2:** Characteristics of the diabetes patients (%)

Characteristics	Male N = 104	Female N = 93	p-value Chi-square
Type of diabetes			0.75
Type 1	7.2	8.1	
Type 2	92.8	91.9	
Duration of diabetes (since first diagnosis)			0.23
< 1 year	8.2	3.8	
2–5 years	34.1	31.7	
6–10 years	33.7	39.8	
> 10 years	24.0	24.7	
Complications			<0.001
None	58.7	30.1	
Major one			
Retinopathy	10.6	17.7	
Nephropathy	3.8	9.7	
Neuropathy	4.3	9.7	
Diabetic foot	4.8	12.9	
Two or more	17.8	19.9	
Treatment for diabetes			0.014
Pills	54.8	51.1	
Insulin	15.9	18.8	
Pills + Insulin	16.8	25.3	
Diet only	12.5	4.8	

**Table 3:** Crude domain scores of WHOQOL-BREF for diabetic patients and controls (mean scores and 95% confidence intervals)

Domain	Diabetes patients	controls
Physical health	36.7 (33.7; 39.6)	75.9 (73.0; 78.8)
Psychological	34.8 (31.8; 37.8)	70.0 (66.7; 72.7)
Social relationships	52.4 (49.7; 55.1)	71.4 (68.7; 74.1)
Environment	23.4 (21.0; 25.9)	36.2 (33.7; 38.6)

**Table 4:** Differences in domain scores associated with determinants of HRQOL (negative numbers mean reduction of HRQOL, positive numbers an improvement in comparison to the references group, adjusted for all other variables in the table, presented is mean difference and its 95% confidence interval, all included interactions were significant at the level of p < 0.05 in multivariate evaluation)

	Physical health	Psychological	Social relationships	Environment
Effect modification by diabetes				
Diabetes patients				
Male	-36.1 (-47.0; -25.3)	-31.8 (-42.7; -20.9)	-14.2 (-24.9; -3.5)	-1.7 (-11.3; 7.9)
Female	-45.2 (-53.4; -37.0)	-45.4 (-53.7; -37.2)	-19.5 (-27.6; -11.5)	-12.3 (-19.6; -5.1)
Controls				
Male	-0.5 (-5.7; 4.7)	-2.0 (-7.2; 3.2)	-1.6 (-12.7; -3.4)	-1.4 (-6.0; 3.2)
Female	Reference	Reference	Reference	Reference
Diabetes patients				
Age below 50	-26.4 (-37.2; -15.6)	-24.9 (-35.8; -14.1)	-8.7 (-19.4; 1.9)	-6.8 (-16.4; 2.7)
50 or more	-45.2 (-53.4; -37.0)	-45.4 (-53.7; -37.2)	-19.5 (-27.6; -11.5)	-12.3 (-19.6; -5.1)
Controls				
Age below 50	-2.2 (-9.5; 5.1)	-3.5 (-10.9; 3.8)	-5.3 (-12.5; 1.9)	3.1 (-3.3; 9.6)
50 or more	Reference	Reference	Reference	Reference
Diabetes patients				
5 or less depending persons	-53.8 (-64.8; -42.8)	-51.4 (-62.4; -40.3)	-28.9 (-39.7; -18.1)	-23.3 (-33.1; -13.6)
6 or more	-45.2 (-53.4; -37.0)	-45.4 (-53.7; -37.2)	-19.5 (-27.6; -11.5)	-12.3 (-19.6; -5.1)
Controls				
5 or less depending persons	4.8 (-1.5; 11.1)	6.7 (0.4; 13.0)	4.0 (-2.2; 10.2)	11.7 (6.1; 17.2)
6 or more	Reference	Reference	Reference	Reference
Other variables				
Younger refugees (age below 50)				
Income below 200	-21.5 (-30.2; -12.8)	-23.4 (-32.1; -14.7)	-15.7 (-24.3; -7.2)	-16.1 (-23.7; -8.4)
Income above 200	-2.2 (-9.5; 5.1)	-3.5 (-10.9; 3.8)	-5.3 (-12.5; 1.9)	3.1 (-3.3; 9.6)
Older refugees (age of 50 or more)				
Income below 200	-10.4 (-15.3; -5.5)	-8.9 (-13.8; -4.0)	-5.5 (-10.3; -0.7)	-9.2 (-13.5; -4.9)
Income above 200	Reference	Reference	Reference	Reference
Younger refugees (age below 50)				
5 or less depending persons	9.7 (-0.5; 19.9)	4.8 (-5.5; 15.0)	7.7 (-2.3; 17.7)	7.0 (-2.0; 16.0)
6 or more	-2.2 (-9.5; 5.1)	-3.5 (-10.9; 3.8)	-5.3 (-12.5; 1.9)	3.1 (-3.3; 9.6)
Older refugees (age of 50 or more)				
5 or less depending persons	4.8 (-1.5; 11.1)	6.7 (0.4; 13.0)	4.0 (-2.2; 10.2)	11.7 (6.1; 17.2)
6 or more	Reference	Reference	Reference	Reference
Marital status				
Other	-4.0 (-8.8; 0.8)	-6.4 (-11.2; -1.7)	-8.0 (-12.7; -3.4)	-2.9 (-7.1; 1.3)
Married	Reference	Reference	Reference	Reference
